# Supplementary material for: CDK9 interacts with a RanGTP–importin-β complex to regulate erythroid enucleation
Source: J Cell Sci. 2026 Apr 9;139(12):jcs264385. doi: 10.1242/jcs.264385 (PMC13091499; doi:10.1242/jcs.264385)
Supplement: Supplementary information [file joces-139-264385-s1.pdf]

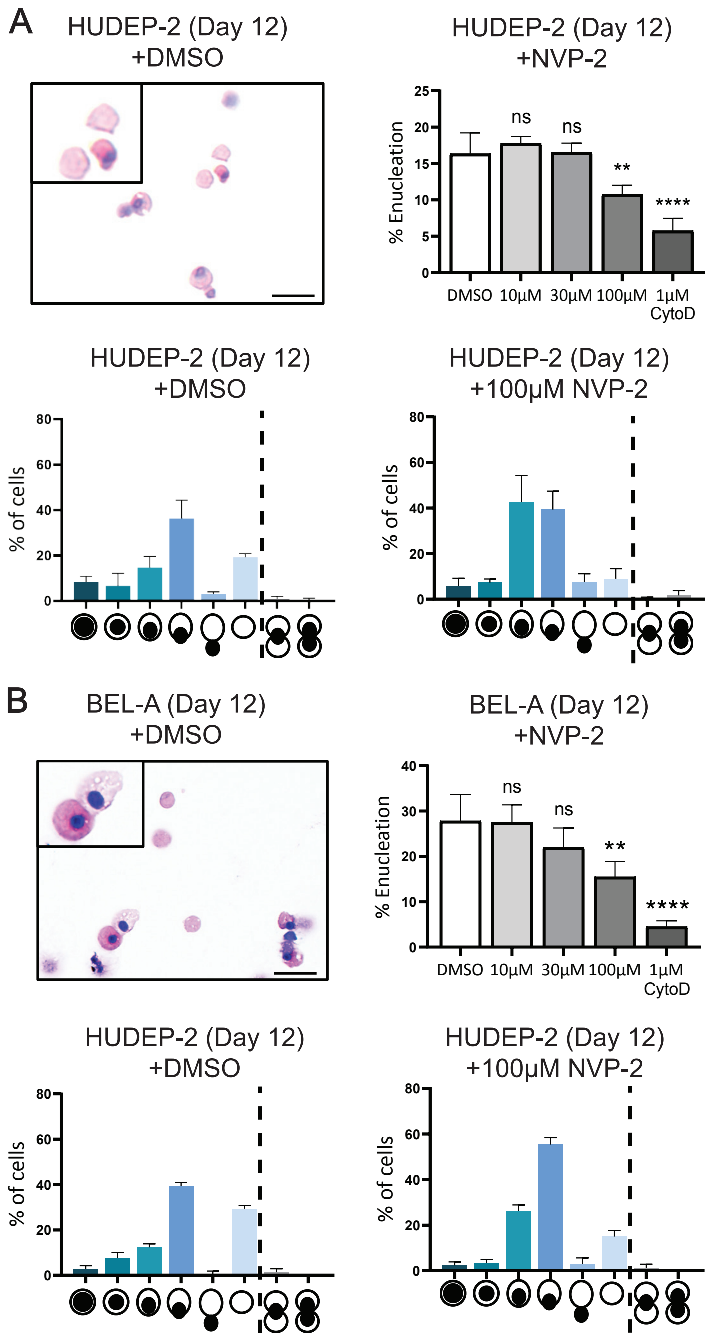

Fig. S1. NVP-2 blocks enucleation of HUDEP-2 and BEL-A cells. (A) Quantification of enucleation of differentiated HUDEP-2 cells (Day 12) following 24-hour treatment with NVP-2. DMSO (vehicle control) and cytochalasin D (positive control) are included, in addition to cytospin rapid diff staining of DMSO control cells and phenotype analysis of DMSO control and NVP- 2 treated cells. n = 205 cells counted (DMSO) and 186 cells counted (NVP-2). (ns = not significant, $^{**}p < 0.01$, $^{****}p < 0.0001$; one-way ANOVA with Dunnett's multiple comparisons test). Scale bar = 10 μm. **(B)** Quantification of enucleation of differentiated BEL-A cells (Day 12) following 24-hour treatment with NVP-2. DMSO (vehicle control) and cytochalasin D (positive control) are included, in addition to cytospin rapid diff staining of DMSO control cells and phenotype analysis of DMSO control and NVP-2 treated cells. n = 235 cells counted (DMSO) and 168 cells counted (NVP-2). (ns = not significant, $^{**}p < 0.01$, $^{****}p < 0.0001$; one-way ANOVA with Dunnett's multiple comparisons test). Scale bar = 10 μm.

Journal of Cell Science • Supplementary information

Supplemental Figure 2
Newton et al (2025)

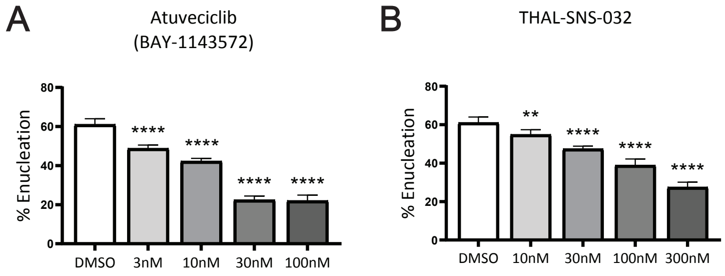

Fig. S2. CDK9 inhibitors Atuveciclib and THAL-SNS-032 cause a dose dependent arrest of mouse erythroblast enucleation. (A) Quantification of enucleation of mouse orthochromatic erythroblasts following treatment with Atuveciclib (BAY1143572) for 12 hours. DMSO (vehicle control) is included. n = 4 replicates across 2 independent experiments. (****p < 0.0001; one-way ANOVA with Dunnett's multiple comparisons test). **(B)** Quantification of enucleation of mouse orthochromatic erythroblasts following treatment with THAL-SNS-032 for 12 hours. DMSO (vehicle control) is included. n = 4 replicates across 2 independent experiments. (**p < 0.01, ****p < 0.0001; one-way ANOVA with Dunnett's multiple comparisons test).

Journal of Cell Science • Supplementary information

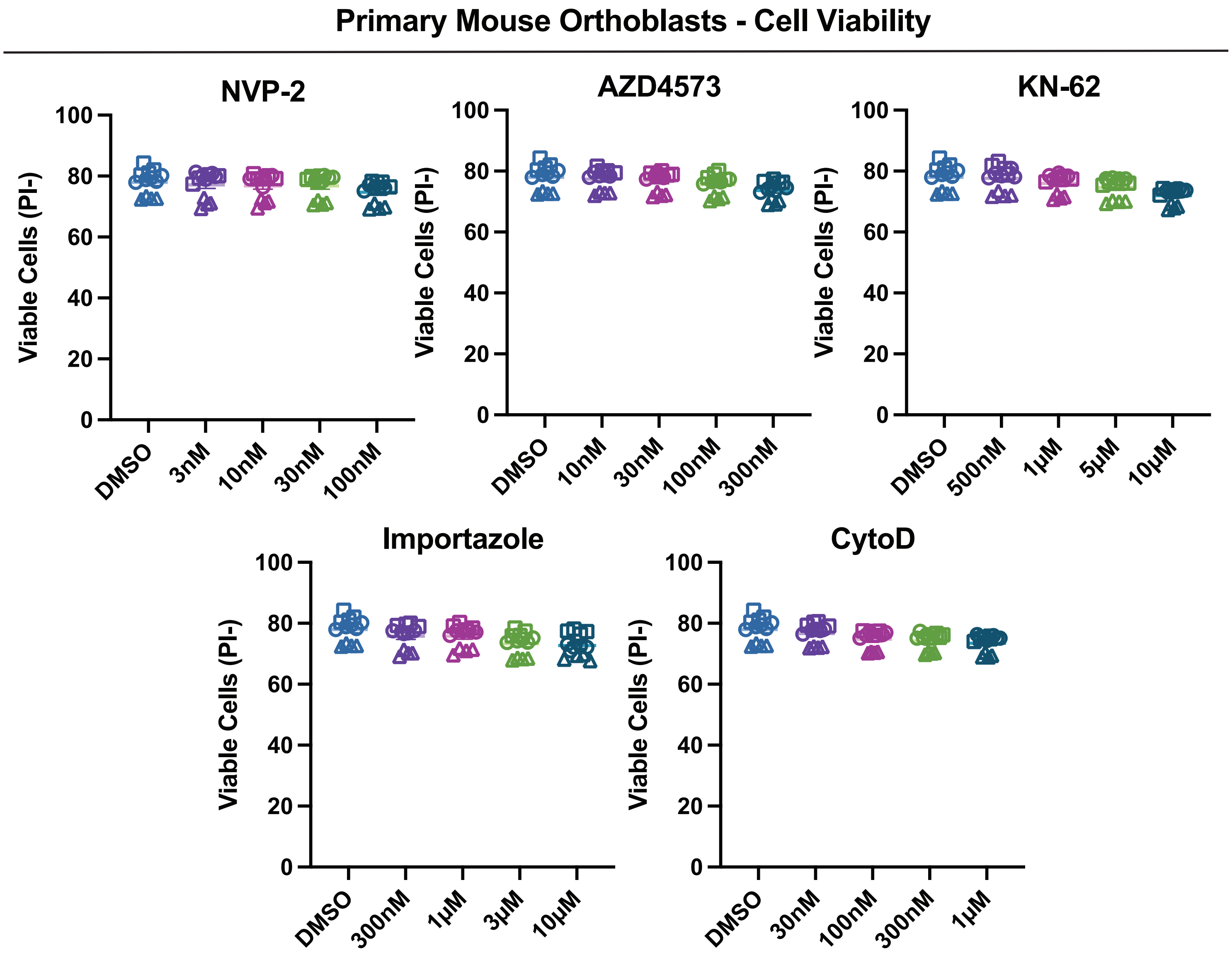

Fig. S3. Cell viability assessment of primary orthochromatic erythroblasts following treatment with pharmacological inhibitors. Primary mouse orthochromatic erythroblasts were treated with various doses of CDK9 inhibitors NVP-2 and AZD4573, CaMKII inhibitor KN-62, importin-β inhibitor Importazole and the actin-polymerisation inhibitor Cytochalasin D (CytoD) for 12 hours and were assessed for propidium iodide (PI) staining. n = 4 technical replicates across 3 independent experiments (denoted by square, circle and triangle data points).

Journal of Cell Science • Supplementary information

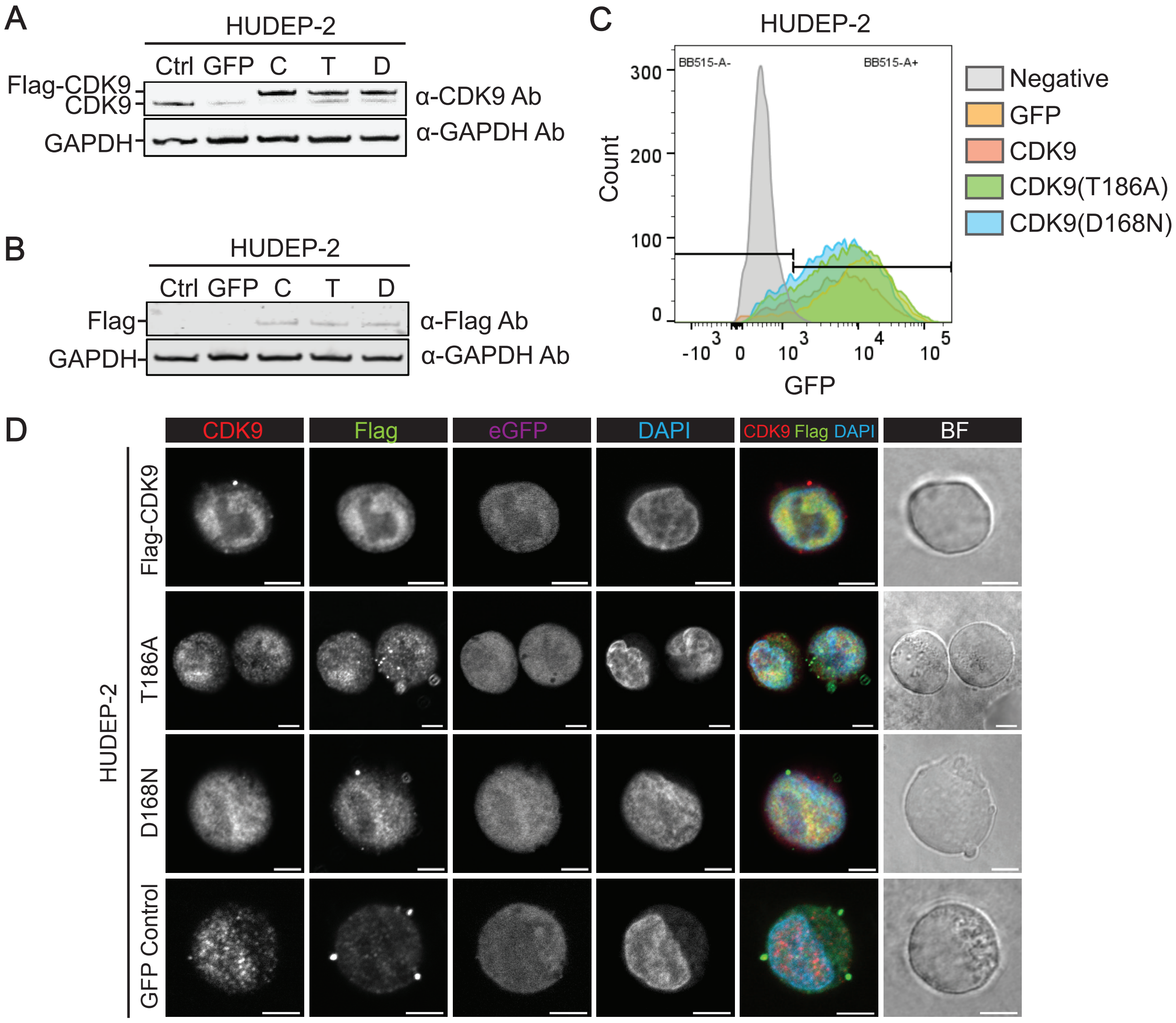

Fig. S4. Characterisation of HUDEP-2 CDK9 overexpression cell lines. (A) Western blot of HUDEP-2 cell lines, including the GFP vector control, CDK9, CDK9-T186A and CDK9-D168N overexpression lines. Bands can be detected for Flag-CDK9 in the three CDK9 overexpression lines. GAPDH is shown as a loading control. **(B)** Western blot of HUDEP-2 cell lines, including the GFP vector control, CDK9, CDK9-T186A and CDK9-D168N overexpression lines. Bands can be detected for Flag in the three CDK9 overexpression lines. GAPDH is shown as a loading control. **(C)** Flow cytometry histogram showing GFP expression in HUDEP-2 cell lines, including the GFP vector control, CDK9, CDK9-T186A and CDK9-D168N overexpression lines compared to negative control HUDEP-2 cells. Propidium iodide was used to gate for only viable cells. **(D)** Immunofluorescence confocal microscopy of undifferentiated HUDEP-2 cells transduced to overexpress CDK9, CDK9-T186A, CDK9-D168N or GFP only and stained for CDK9, Flag, GFP and DAPI for nuclei. Transduced cells expressing low levels of eGFP are shown in comparison to the wildtype. Brightfield (BF) and eGFP are excluded from the merge. Yellow colour depicts co-localisation of CDK9 and Flag. All scale bars = 5 µm.

Journal of Cell Science • Supplementary information

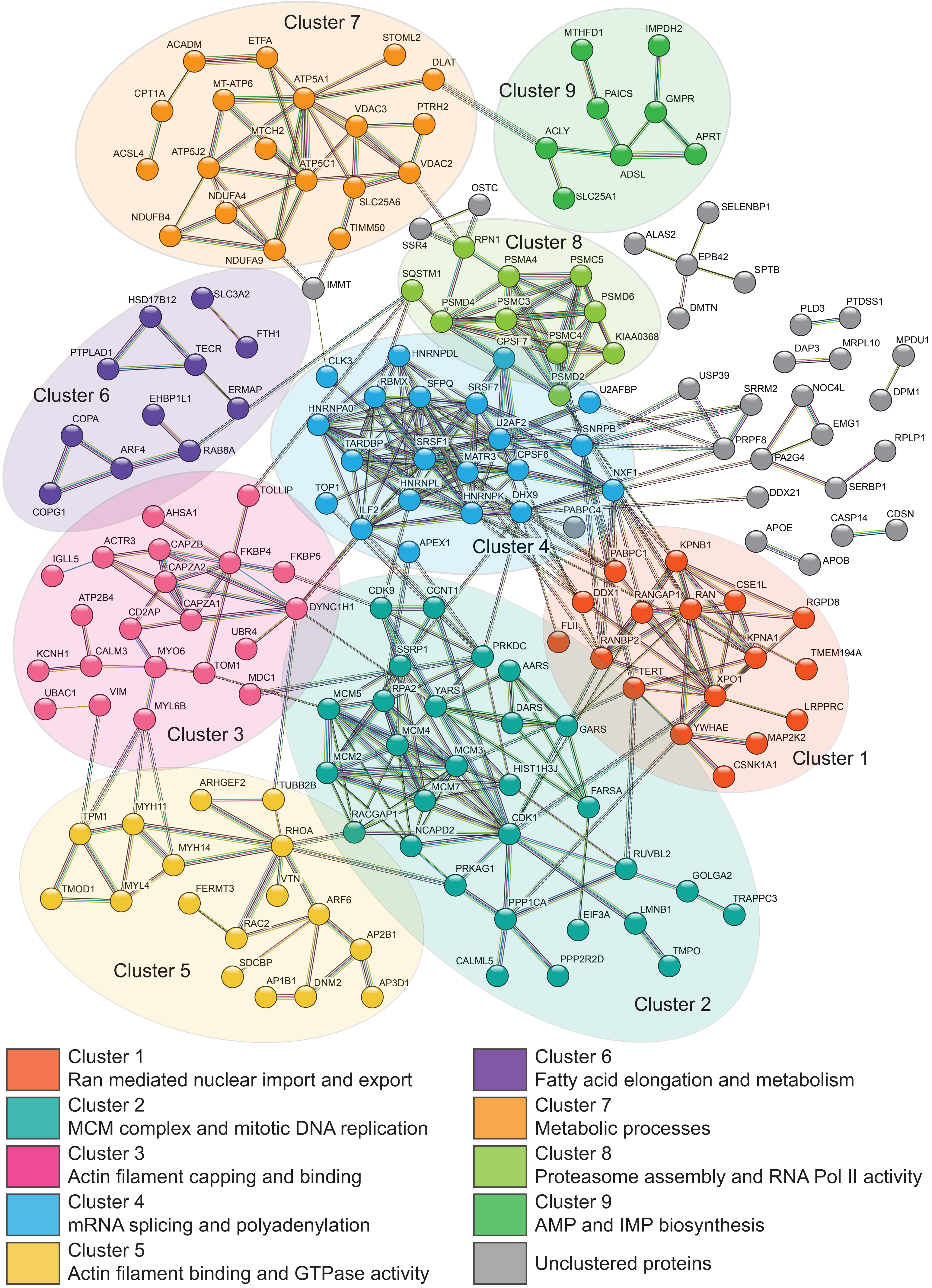
Cluster 7
ACADM
ETFA
STOML2
DLAT
CPT1A
MT-ATP6
ATP5A1
VDAC3
PTRH2
MTCH2
ACSL4
ATP5J2
ATP5C1
VDAC2
NDUFA4
SLC25A6
NDUFB4
TIMM50
NDUFA9
Cluster 9
MTHFD1
IMPDH2
PAICS
GMPR
ACLY
APRT
ADSL
SLC25A1
OSTC
SELENBP1
SSR4
RPN1
Cluster 8
ALAS2
EPB42
SPTB
PSMA4
PSMC5
SQSTM1
IMMT
PSMD4
PSMC3
PSMD6
DMTN
PLD3
PTDSS1
PSMC4
KIAA0368
CPSF7
DAP3
MRPL10
MPDU1
HSD17B12
SLC3A2
FTH1
PTPLAD1
TECR
ERMAP
Cluster 6
EHBP1L1
COPA
ARF4
RAB8A
COPG1
HNRNPDL
CLK3
RBMX
SFPQ
SRSF7
PSMD2
U2AFBP
USP39
SRRM2
NOC4L
DPM1
HNRNPA0
U2AF2
SNRPB
EMG1
TARDBP
SRSF1
MATR3
CPSF6
PRPF8
PA2G4
RPLP1
NXF1
TOP1
HNRNPL
HNRNPK
DHX9
DDX21
SERBP1
TOLLIP
ILF2
PABPC4
AHSA1
CASP14
CDSN
APOE
ACTR3
CAPZB
APEX1
Cluster 4
KPNB1
IGLL5
FKBP4
FKBP5
PABPC1
APOB
CAPZA2
CSE1L
ATP2B4
CAPZA1
CDK9
CCNT1
DDX1
RANGAP1
RAN
RGPD8
CD2AP
DYNC1H1
FLII
UBR4
RANBP2
KPNA1
KCNH1
CALM3
MYO6
SSRP1
PRKDC
TMEM194A
TOM1
AARS
TERT
XPO1
UBAC1
VIM
MDC1
MCM5
RPA2
YARS
DARS
LRPPRC
MYL6B
YWHAE
Cluster 3
MCM4
GARS
MAP2K2
MCM3
Cluster 1
MCM2
HIST1H3J
CSNK1A1
ARHGEF2
TUBB2B
MCM7
FARSA
TPM1
MYH11
RACGAP1
RHOA
NCAPD2
CDK1
MYH14
RUVBL2
GOLGA2
PRKAG1
TMOD1
MYL4
FERMT3
VTN
ARF6
PPP1CA
TRAPPC3
EIF3A
LMNB1
RAC2
AP2B1
TMPO
SDCBP
CALML5
PPP2R2D
Cluster 5
AP1B1
DNM2
AP3D1
Cluster 2
Cluster 1
Ran mediated nuclear import and export
Cluster 2
MCM complex and mitotic DNA replication
Cluster 3
Actin filament capping and binding
Cluster 4
mRNA splicing and polyadenylation
Cluster 5
Actin filament binding and GTPase activity
Cluster 6
Fatty acid elongation and metabolism
Cluster 7
Metabolic processes
Cluster 8
Proteasome assembly and RNA Pol II activity
Cluster 9
AMP and IMP biosynthesis
Unclustered proteins

Fig. S5. Interactome of identified proteins in HUDEP-2 CDK9 overexpression cell lines. STRING analysis of proteins identified across all HUDEP-2 overexpression lines. Proteins were considered an interactor of CDK9 if identified in all 3 replicates and were ≥3 fold enriched compared to the IgG control. Clusters were identified using STRING analysis of all proteins identified in overexpression lines (243 total proteins), with high confidence (0.7) for interaction score, with unconnected nodes removed. Clustering was performed using kmeans clustering into 11 groups. Aqua lines represent known interactions from curated databases, purple lines represent experimentally determined known interactors. Green, red, and blue lines represent predicted interactors, yellow lines represent text mining identification and black lines represent known co-expression. Dotted lines represent connections between clusters. See the supplementary file for a complete list of identified proteins and corresponding abundances.

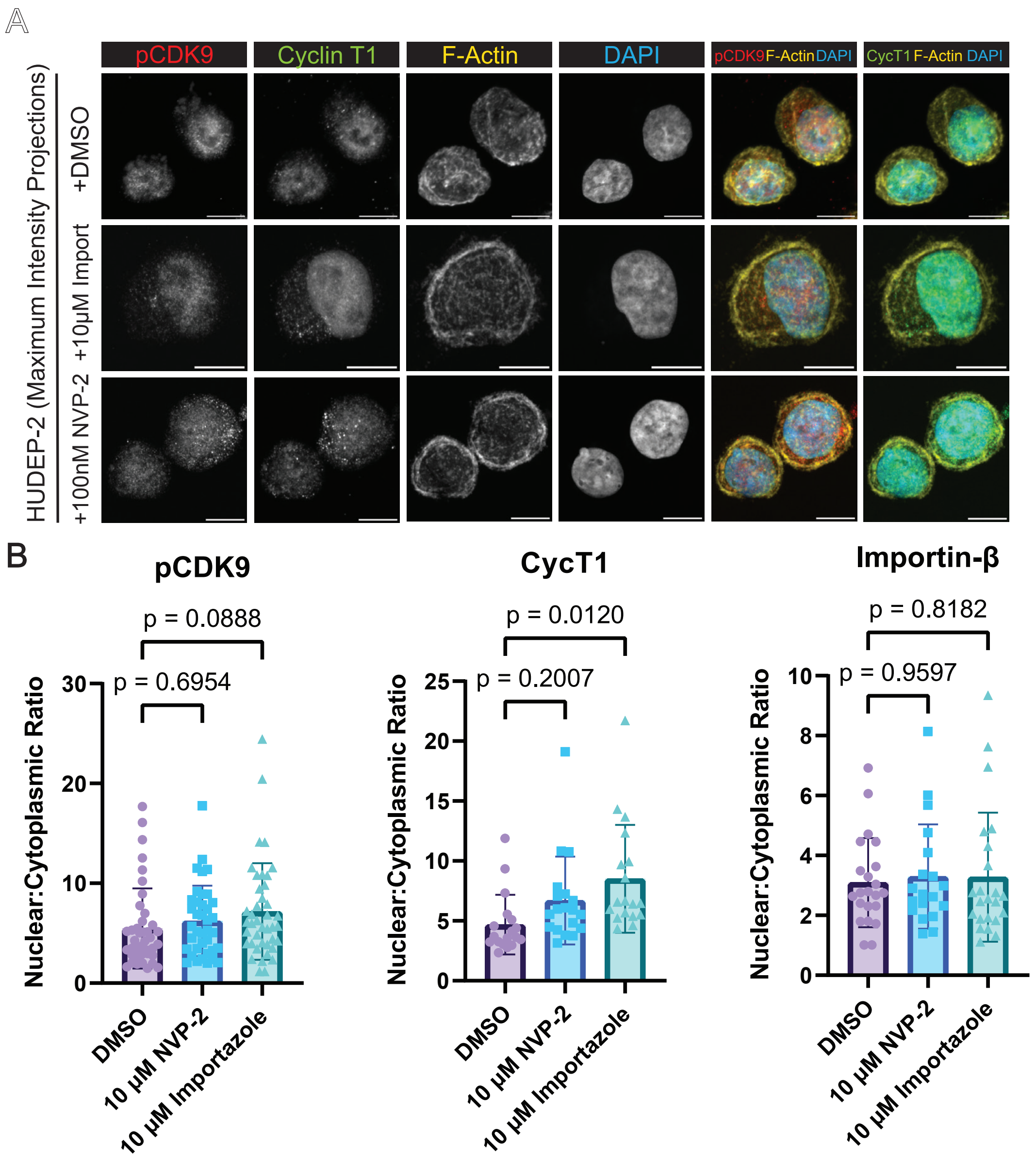

Fig. S6. Importazole alters the nucleocytoplasmic ratio of Cyclin T1 but not phosphorylated CDK9. (A) Immunofluorescence confocal microscopy of undifferentiated (Day 0) HUDEP-2 cells stained for phospho-CDK9(Thr186), cyclin T1, F-actin and DAPI. Merges showing pCDK9 and cyclin T1 alongside F-actin are shown. All scale bars = 5 µm. **(B)** Analysis of nuclear to cytoplasmic ratio of pCDK9, cyclin T1 and importin-β in fixed undifferentiated (Day 0) HUDEP-2 cells following overnight treatment with 10µM NVP-2, 10µM Importazole or DMSO vehicle control. Each point represents an individual cell. pCDK9 DMSO n = 40, NVP-2 n = 38, Importazole n = 41; CycT1 DMSO n = 18, NVP-2 n = 19, Importazole n = 19; Importin-β DMSO n = 22, NVP-2 n = 19, Importazole n = 23. (p values are shown for each comparison; one-way ANOVA with Dunnett's multiple comparisons test).

Journal of Cell Science • Supplementary information

Table S1. Complete list of all antibodies and reagents used in this study. Information in this table includes antibody clone identifiers and concentrations used in this study, and whether antibodies have been validated by the supplier, in addition to the company and location of the manufacturer of all reagents utilised.

Available for download at
https://journals.biologists.com/jcs/article-lookup/doi/10.1242/jcs.264385#supplementary-data

Table S2. Spreadsheet containing CDK9 Co-IP-MS raw data (proteomics searches) and subsequent data used for analysis in this study. This excel file contains raw data (proteomics searches) for HUDEP-2 day 0 (Sheet 1), raw data (proteomics searches) for HUDEP-2 day 6 (Sheet 2), day 6 overexpression analysis (Sheet 3), day 6 wild-type (WT) analysis (Sheet 4), day 0 wild-type (WT) analysis (Sheet 5), and CDK9 overexpression data used to generate the heatmaps in Figure 4 (Sheet 6). Full raw mass spectrometry data have also been deposited to the ProteomeXchange Consortium via PRIDE partner repository with the dataset identifier PXD072077.

Available for download at
https://journals.biologists.com/jcs/article-lookup/doi/10.1242/jcs.264385#supplementary-data
